# Energy metabolism and obesity stratified by BMI: impact on lipid oxidation, a cross-sectional observational study

**DOI:** 10.3389/fnut.2025.1701686

**Published:** 2025-11-27

**Authors:** Lorena de-Medeiros, Rocío San Martin, Gizela Junqueira, Joyce de Oliveira, Silvana Trinidad Trunce-Morales, Carlos Nogueira-de-Almeida, Julio Sérgio Marchini

**Affiliations:** 1 Departamento de Clínica Médica, Faculdade de Medicina de Ribeirão Preto, Universidade de São Paulo, São Paulo, Brazil; 2 Departamento de Salud, Universidad de Los Lagos, Osorno, Chile; 3 Departamento de Nutrição e Metabolismo, Faculdade de Medicina de Ribeirão Preto, Universidade de São Paulo, São Paulo, Brazil; 4 Departamento de Medicina, Universidade Federal de São Carlos, São Carlos, Brazil

**Keywords:** obesity, resting metabolic rate, energy expenditure, body mass index, indirect calorimetry, energy metabolism, resting energy expenditure

## Abstract

**Introduction:**

Obesity is a complex disorder of nutritional-metabolic factors, involving, among others, the environment, mental health, and physical activity levels. Biological and social aspects are fundamental to understanding the genesis and progression of obesity. Energy metabolism, particularly resting energy expenditure (REE) and substrate oxidation, plays a key role in maintaining energy balance. REE reflects the energy required to sustain vital functions such as cellular metabolism, organ function, and homeostasis. Variations in body mass index (BMI) are associated with differences in REE and substrate oxidation, which may contribute to the metabolic predisposition to obesity. Based on this, we hypothesize that in obesity, the energy metabolism is characterized by reduced lipid oxidation.

**Objective:**

To explore the relationship between energy metabolism parameters and predisposition to obesity.

**Methods:**

An observational, cross-sectional study was conducted among 216 adult women (≥18 years), non-hospitalized and free of chronic diseases, with different BMI classifications. Between 2017 and 2024, energy metabolism assessments were performed using indirect calorimetry, with direct measurement of oxygen consumption (VO₂) and carbon dioxide production (VCO₂) at rest, and substrate oxidation estimated from respiratory quotient analysis.

**Results:**

Women with obesity (BMI ≥ 30 kg/m^2^) showed a significantly higher rate of REE and lipid oxidation compared with women with normal BMI (<25 kg/m^2^) (*p* < 0.05). Women with obesity oxidize more lipids than women without obesity (*p* < 0.05).

**Conclusion:**

In adult women, obesity is associated with elevated resting energy expenditure and lipid oxidation, which demonstrated discriminatory value in distinguishing the obese state from non-obese women. These results underscore the relevance of energy metabolism parameters in understanding the metabolic adaptations linked to higher BMI.

Highlights

- Obesity is associated with increased resting energy expenditure in women.
- Higher lipid oxidation at rest suggests metabolic adaptation to adiposity.
- Resting energy expenditure discriminates obesity better than lipid or CHO oxidation.
- Findings challenge the assumption that obesity is sustained by lower REE.

## 1 Introduction

Obesity is a multifactorial and chronic disease characterized by an imbalance between energy intake and expenditure, involving complex interactions among nutritional, metabolic, psychosocial, and behavioral factors (1–6). Understanding these interrelated mechanisms is essential to elucidate the genesis and progression of obesity, emphasizing the importance of evaluating energy metabolism in affected individuals (1–4, 7).

Resting energy expenditure (REE) represents the minimum amount of energy required to maintain vital physiological functions under resting conditions (8). It is a fundamental determinant of total energy requirements and plays a pivotal role in human physiology, nutrition, and metabolic health. Variations in REE reflect individual differences in energy metabolism and can influence weight regulation and body composition (8, 9).

Obesity is associated with alterations in metabolic efficiency, substrate utilization, and body composition (2, 10–12). A chronic positive energy balance contributes to weight gain, while the proportion of metabolically active tissues—particularly fat-free mass—directly affects REE and substrate oxidation (10, 13). Thus, evaluating the interplay between REE, lipid and carbohydrate oxidation, and adiposity provides valuable insights into metabolic adaptations related to obesity.

Despite its clinical relevance, few studies have investigated differences in REE and substrate oxidation across body mass index (BMI) categories in women. Identifying factors that modulate energy metabolism may improve personalized dietary prescriptions and guide metabolic rehabilitation strategies (9, 14).

Therefore, this study aimed to compare energy metabolism parameters, including REE and substrate oxidation, among women classified into different BMI categories.

## 2 Methods

### 2.1 Data sources and extraction

The study was approved by the Ethics Committee of the Clinical Hospital of Ribeirão Preto of Universidade de São Paulo, São Paulo, Brazil.

A cross-sectional observational study was conducted of the Clinical Hospital of Ribeirão Preto of Universidade de São Paulo (São Paulo, Brazil) with non-hospitalized adult women classified into different BMI categories (15): normal weight (18.5–24.9 kg/m^2^), overweight (25.0–29.9 kg/m^2^), and obesity (≥30 kg/m^2^).

Eligibility criteria included women (biological female sex) aged 18 years or older, able to undergo indirect calorimetry assessment of the Clinical Hospital of Ribeirão Preto of Universidade de São Paulo. Exclusion criteria were hospitalization and critical chronic illness.

The minimum sample size was determined using the equation proposed by Agranonik and Hirakata (16). The sample size calculation for comparing two proportions was applied, considering independent variables and known epidemiological proportions of obesity among adult women (16). The sample size of 49 subjects in each group (obese and non-obese) was determined based on power analysis, aiming for a statistical power of ≥0.8 at an *α* level of 0.05, with value of the *Cα, power* of 7.9 (16). For this complementary analysis, the sample size calculation was performed using R software (pwr package), based on a two-sample Student’s *t*-test. Considering a two-sided test, an effect size of 0.8 was assumed, with REE means of 1345.1 ± 178.5 kcal for non-obese women (17) and 1597.04 ± 327.83 kcal for obese women (18), as indicated in the literature. The calculation, based on the mean differences (19), used a significance level of 0.05 and 95% power, yielding a required sample size of 42 participants per group.

All participants who met the eligibility criteria and agreed to participate (*n* = 216) were included in the final analyses. As this was a single time-point cross-sectional study without follow-up, there were no losses to follow-up or participant withdrawals.

Body weight was measured to the nearest 0.050 kg using a calibrated electronic scale (WELMY^®^ W300, Brazil) with a minimum capacity of 1 kg and a maximum capacity of 300 kg. Height was measured to the nearest 0.1 cm using a stadiometer (WELMY^®^ W300, Brazil). Body mass index (BMI) was subsequently calculated as weight (kg) divided by height squared (m^2^).

### 2.2 Indirect calorimetry

Data collection was performed at a single point in time, with no longitudinal follow-up. Energy metabolism assessments were conducted using indirect calorimetry (QUARK-RMR COSMED^®^, Rome, Italy), with direct measurement of oxygen consumption (VO₂) and carbon dioxide production (VCO₂) at rest, allowing for the analysis of substrate oxidation through the calculation of the respiratory quotient proposed by Martin (8) and Frayn (20).

Measurements were carried out under standardized resting conditions, in the supine position, with participants fasting for 12 h, instructed to refrain from physical activity, and to avoid the consumption of caffeine-based beverages, black tea, or alcoholic drinks within 24 h prior to the assessment, and without menstrual period, in a quiet and climate-controlled environment maintained between 21 and 24 °C (8). REE was measured, which was performed after a resting period of 15–20 min (8). During the measurement, participants were awake while lying on a bed in a quiet room. The volume of oxygen inspired and carbon dioxide exhaled were measured every minute.

VO₂ and VCO₂ were recorded for 15–30 min. Data obtained during the first 5 min were discarded to ensure the participant reached a steady state (CV < 10%). The recorded values were analyzed according to Weir for REE estimation and Frayn for the calculation of energy substrate oxidation (8, 20, 21).

### 2.3 Statistical analysis

Data normality was assessed using the Shapiro–Wilk test. As some variables showed a normal distribution while others did not, the mean ± standard deviation and the median (min–max) are presented, providing a more comprehensive description of central tendency and data dispersion.

Clinical data and prevalence of obesity were compared across different BMI classification groups (normal weight, overweight, and obesity). Continuous variables were compared using one-way analysis of variance (ANOVA), followed by Tukey’s *post-hoc* test for multiple comparisons, while categorical variables were compared using the chi-square test.

To address the main objective of the study—examining the association between REE, substrate oxidation rates, and obesity status—a multivariable logistic regression model was constructed using obesity (1 = obese, 0 = non-obese) as the dependent variable. Independent variables included REE, carbohydrate and lipid oxidation rates, and respiratory quotient (RQ). Adjusted odds ratios (ORs) with 95% confidence intervals (CIs) were reported.

Additionally, linear regression analyses were performed to explore the relationship between BMI (continuous outcome) and metabolic variables.

The discriminative ability of substrate oxidation parameters and REE to predict obesity was further evaluated using receiver operating characteristic (ROC) curves, and the DeLong test was used to compare the areas under the curves (AUCs).

All analyses were conducted using R statistical software (v4.0.3),^1^ with statistical significance set at *p* < 0.05 (two-tailed).

## 3 Results

### 3.1 Patient characteristics based on BMI

A total of 216 women were included in the final analysis. Table 1 shows a summary of the clinical and energy-metabolic characteristics of the participants (*N* = 216) across different BMI categories: normal weight, overweight, and obesity. Although all individuals with obesity were analyzed as a single group, differences in BMI severity were present within this category. Among participants classified within the obesity group (*n* = 151), 87 (57.6%) were categorized as Class I (BMI 30–34.9 kg/m^2^), 56 (37.1%) as Class II (BMI 35–39.9 kg/m^2^), and 8 (5.3%) as Class III (BMI ≥ 40 kg/m^2^), according to the WHO classification (15). An outlier (95.7 kg/m^2^) was also identified, corresponding to a single participant. The distribution of BMI values for all participants, including potential outliers, is shown in Supplementary Figure S1. The data include variables such as age, height, weight, BMI, REE, and carbohydrate (CHO) and lipid oxidation rates, stratified by BMI category.

**Table 1:** Characteristics for the study population, classified by BMI (*N* = 216).

Variable	F (%)/n	Mean ± SD	Median (min—max)	CV
Age (years)		37.7 ± 13.8		0.365
Normal weight	20.83% (45)	31.4 ± 12.4		0.395
Overweight	9.5% (20)	36.0 ± 10.6		0.296
Obesity	69.91% (151)	39.8 ± 14.0		0.352
Weight (kg)	–		84.650 (44.100–245.00)	0.236
Normal weight	20.83% (45)		57.800 (44.100–72.900)	0.109
Overweight	9.25% (20)		75.750 (67.599–92.299)	0.086
Obesity	69.91% (151)		90.099 (70.000–245.000)	0.179
BMI (kg/m^2^)	–		32.7 (18.6–95.7)	0.234
Normal weight	20.83% (45)		21.5 (18.6–24.9)	0.074
Overweight	9.25% (20)		28.4 (25.2–29.8)	0.057
Obesity	69.91% (151)		34.32 (30.0–95.7)	0.165
REE (kcal/day)	–	1,687 ± 338		0.200
Normal weight	20.83% (45)	1,442 ± 144		0,168
Overweight	9.25% (20)	1,642 ± 234		0.142
Obesity	69.91% (151)	1766 ± 339		0.192
CHO oxidation (kcal/day)	–		430 (−782–1823)	1.301
Normal weight	20.83% (45)		529 (−189–1802)	0.168
Overweight	9.25% (20)		438 (−692–1,244)	1.379
Obesity	69.91% (151)		316 (−782–1823)	1.513
CHO oxidation (%)	–		27.89 (−37.56–124.46)	1.218
Normal weight	20.83% (45)		39.07 (−12.72–124.46)	0.753
Overweight	9.25% (20)		25.27 (−35.75–69.52)	1.285
Obesity	69.91% (151)		17.39 (−37.56–108.02)	1.402
Lipid oxidation (kcal/day)		1,215 ± 680		0.560
Normal weight	20.83 % (45)	857 ± 440		0.513
Overweight	9.25 % (20)	1,209 ± 551		0.456
Obesity	69.91 % (151)	1,322 ± 720		0.544
Lipid oxidation (%)	–		69.13 (−16.12–126.91)	0.443
Normal weight	20.83 % (45)		59.26 (−16.12 to 104.99)	0.462
Overweight	9.25 % (20)		71.44 (32.37–125.31)	0.378
Obesity	69.91% (151)		78.40 (−1.60–126.91)	0.438

Values are expressed in absolute and relative frequency. Values CV are expressed as SD/ mean. The distribution of all variables was verified using the Shapiro–Wilk test. Normally distributed variables are expressed in Mean ± SD. Non-normal distributions variables are expressed in Median (min—max). BMI, body mass index; SD, standard deviation; min, minimum; max, maximum; CV, coefficient of variation; F, frequency; m: meters; REE, resting energy expenditure; Kcal, kilocalorie; Kg, kilogram; g, gram; %, percentage; CHO, carbohydrate; RQ, respiratory ratio; VO_2_, volume of inspired oxygen; VCO_2_, volume of expired carbon dioxide.

Participants with obesity showed a significantly higher REE compared to those with normal weight and overweight (*p* < 0.05). Lipid oxidation was higher in participants with obesity than in those with normal weight, (*p* < 0.05).

The coefficient of variation (CV) was calculated for each variable, reflecting data dispersion within each BMI group (Table 1). The highest CV values were observed for carbohydrate and lipid oxidation, indicating high interindividual variability, especially in the obese group.

### 3.2 Independent factors for BMI

Table 2 summarizes the results of the multivariable logistic regression analysis examining the association between BMI and energy metabolism variables. These findings expand on the group comparisons described in section 3.1 by identifying independent associations between body weight status and substrate oxidation profiles.

**Table 2:** Results of multivariable logistic regression analysis for the relationship between BMI.

Variable	BMI	Estimate	*p*-value
REE (kcal/day)		324 kcal/day	
	Normal weight		
	Overweight	200	<0.05
	Obesity	324	<0.001*
Oxidation CHO (kcal/day)		−182 kcal/day	
	Normal weight		
	Overweight	−186	>0.05
	Obesity	−182	<0.05
Lipid oxidation (kcal/day)		465 kcal/day	
	Normal weight		
	Overweight	352	<0.05
	Obesity	465	<0.001*
% CHO		−15.06 %	
	Normal weight		
	Overweight	−16.20	>0.05
	Obesity	−15.06	<0.001*
% Lipid		13.29 %	
	Normal weight		
	Overweight	14.30	>0.05
	Obesity	13.29	<0.001*

*p* < 0.05 compared with normal weight group (); *p* < 0.001 compared with normal weight group (*). Multivariable logistic regression. BMI, body mass index; REE, energy expenditure savings; kcal, kilocalorie; %, percentage; CHO, carbohydrate.

Obesity was associated with a significantly higher REE compared with normal weight (*p* < 0.001). Significant differences were detected between the overweight group and either comparison group (*p* < 0.05), suggesting that the increase in REE becomes more evident at higher degrees of adiposity.

Conversely, carbohydrate oxidation was significantly lower in individuals with obesity (*p* < 0.001) compared with normal weight, while the overweight group showed no significant differences relative to normal weight (*p* > 0.05), suggesting an association between reduced carbohydrate oxidation and obesity rather than overweight.

Similarly, the percentage of carbohydrate oxidation was also significantly reduced in obesity (*p* < 0.001) compared with normal weight. Although the overweight group exhibited a similar reduction, this difference did not reach statistical significance (*p* > 0.05).

In contrast, lipid oxidation showed the opposite trend: both overweight and obese individuals displayed significantly higher lipid oxidation compared with normal-weight participants (*p* < 0.05). The relative contribution of lipids to total energy expenditure was also greater in the obese group (p < 0.001).

### 3.3 Further analysis of predictors of obesity

**Figure 1:**
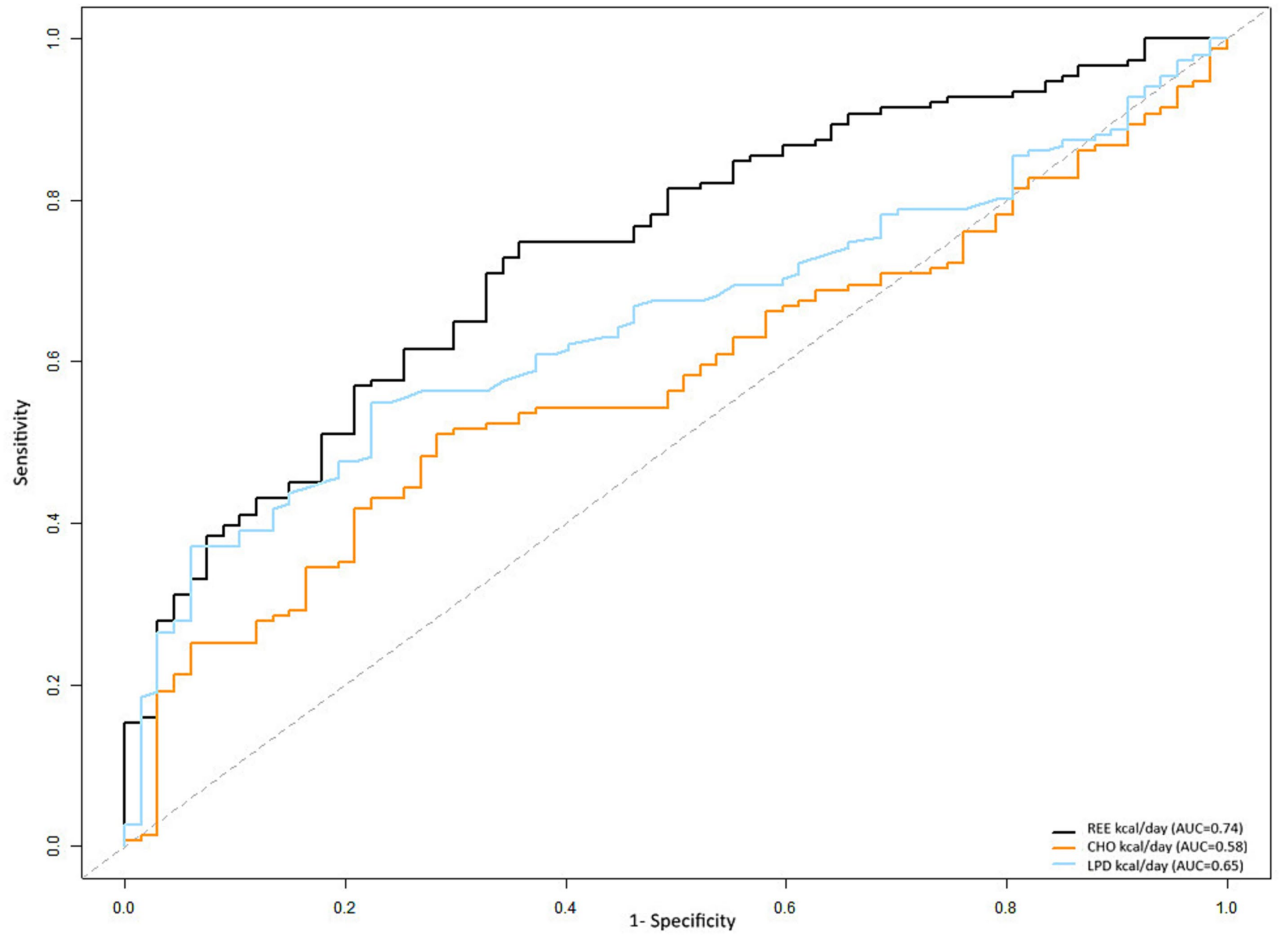
ROC curves of predictor variables for obesity in the study.

The statistical comparison of substrate oxidation variables was performed using ROC curves analysis (Figure 1) to examine their association with obesity. The comparison of ROC curves further demonstrated that REE (kcal/day) exhibited significantly superior discriminatory performance for obesity compared with substrate oxidation variables.

The DeLong test (Table 3) indicated that the AUC for REE was significantly higher than that for carbohydrate oxidation (*p* < 0.05) and lipid oxidation (*p* < 0.05). Thus, the AUC for lipid oxidation was significantly greater than that for carbohydrate oxidation.

**Table 3:** Comparisons of AUCs of ROC curves.

Variables	z	*P*-value**	IC AUC*	Sample estimates: AUC of roc1 AUC of roc2			
				Variables × Variables	REE AUC	CHO AUC	LPD AUC
REE × CHO	3.67	<0.05	0.07–0.25	0.74 0.58	0.74	0.58	–
REE × LPD	2.95	<0.05	0.03–0.15	0.74 0.65	0.74	–	0.65
CHO × LPD	−4.03	<0.05	− 0.11 to −0.04	0.58 0.65	–	0.58	0.65

**DeLong’s test for two correlated ROC curves. *Alternative hypothesis: true difference in AUC is not equal to 0.95 percent confidence interval. Values are presented rounded to two decimal places for clarity. The raw (unrounded) data are available in the Supplementary material. REE, resting energy expenditure; CHO, carbohydrate; LPD, lipid; CI, confidence interval; AUC, Area Under the Curve.

Table 4 presents the results of the logistic regression analysis aimed at identifying predictors of obesity based on different indices of metabolic substrate oxidation, expressed as respiratory quotient for carbohydrates, lipids, and proteins.

**Table 4:** Logistic regression predicting to for obesity.

Variables	OR (95% CI)	*p*-value	Adjusted OR	Adjusted *p*-value
RQ CHO	0.4 (0.03, 4.83)	>0.05	0 (0, 0.12)	<0.05
RQ LPD	0.29 (0.09, 0.89)	<0.05	0.1 (0.03, 0.37)	<0.05
RQ PRO	0.33 (0.05, 2.33)	>0.05	0.04 (0, 0.34)	<0.05
RQ MIX	0.23 (0.07, 0.72)	<0.05	0.01 (0, 0.08)	<0.05

Multiple R-squared. Adjusted ORs and *p*-values were obtained from a multiple (multivariable) logistic regression model including all variables listed in the table simultaneously. RQ, respiratory ratio; OR, odds ratio; CI, confidence interval. REE, resting energy expenditure; RQ CHO, Respiratory Quotient for Carbohydrates; RQ LPD, Respiratory Quotient for Lipids; RQ PRO, Respiratory Quotient for Proteins; RQ MIX, Respiratory Quotient for Mixed Substrates.

The adjusted analysis indicated a significant risk reduction (*p* < 0.05).

Lipid oxidation emerged as a variable associated with obesity in both crude and adjusted models. The adjusted odds ratio (OR = 0.1; *p* < 0.05) indicated that higher lipid oxidation was related to obesity.

Protein oxidation was also identified as an important predictor in the adjusted model, with an adjusted OR of 0.04 (*p* < 0.05). However, this association was not sufficiently robust to be considered a reliable risk factor for obesity (95% CI: 0.05–2.33).

Mixed macronutrient oxidation was significantly associated with a reduced risk of obesity, with an extremely low adjusted odds ratio and a highly significant *p*-value (OR = 0.01; *p* < 0.05).

## 4 Discussion

### 4.1 Lipid oxidation

One of the most intriguing findings of the present study was that, contrary to prevailing theories such as the fuel partitioning theory and the concept of “metabolic inflexibility” (22–24), women with obesity exhibited a higher absolute rate of lipid oxidation at rest compared with their normal-weight counterparts, even in the absence of dietary intervention or weight loss. This result challenges the predominant view that obesity is associated with regulated fat mobilization and reduced metabolic flexibility, suggesting instead that, under certain physiological conditions, excess adipose tissue and the greater availability of free fatty acids may induce a metabolic adaptation characterized by increased lipid oxidation. However, such an adaptation does not necessarily confer protection against body fat accumulation, since a positive energy balance can still be sustained if caloric intake exceeds energy expenditure (25) or if variations in energy absorption and excretion occur (26).

We did not identify other studies that specifically examined the relative proportion of fat oxidation required to meet resting energy expenditure, in which this proportion was significantly lower in normal-weight women than in those with obesity. To date, there is no robust body of clinical trial evidence addressing lipid oxidation in obesity among sedentary adults, particularly about its contribution to energy expenditure.

Some studies have investigated the modulation of lipid oxidation in obesity in response to vigorous physical activity; for example, Colpitts et al. (27) and Güzel et al. (28) examined postmenopausal women with obesity, focusing on changes during submaximal exercise. Their findings showed no significant alterations in lipid oxidation at rest. Other studies have explored lipid oxidation in different contexts and populations, with some showing no significant effects (29, 30).

In summary, the higher resting lipid oxidation observed in women with obesity may be related to the increased volume of adipose tissue, which, despite its relatively low metabolic activity per unit mass (31), can contribute to greater circulating free fatty acid availability and, consequently, enhance basal lipid oxidation (22, 23, 32).

Overall, our findings indicate significant metabolic alterations, as also reflected by the elevated energy expenditure observed in obesity. These results can be partly explained by differences in body composition, since metabolically active tissues such as fat-free mass influence resting energy expenditure and substrate oxidation. Moreover, previous studies have shown that metabolic rate may also vary according to body fat distribution, which affects the proportion and activity of these metabolically active tissues (10, 13).

Our findings may reflect a chronic metabolic adaptation to the higher adipose tissue content and the increased availability of free fatty acids, potentially indicating an adaptive response aimed at reducing the risk of further fat accumulation or preventing the progression of obesity (33). It is also important to consider that some individuals with obesity may exhibit a greater absolute capacity for lipid oxidation, while their relative oxidation rate may not be as elevated due to the increase in overall energy expenditure (34).

These issues highlight the complexity of the interactions between body composition and substrate metabolism, particularly in studies involving women, in whom regulatory mechanisms appear to be less dependent on classical hormonal signals and more directly influenced by total adipose mass, in intervention-specific and sex-specific (32).

### 4.2 Resting energy expenditure

The second most important finding of this study—the higher REE in obesity compared with normal weight—reflects metabolic changes associated with increased body mass, which includes both adipose tissue and a proportional increase in fat-free mass (muscle and organs). Obesity is generally associated with higher energy expenditure due to the concomitant rise in lean mass and the additional energy required to carry and move excess body weight (22, 31, 34, 35). Thus, the higher REE observed may result from a greater energy demand to sustain a body with increased tissue mass, requiring enhanced organ and tissue maintenance even at rest (35).

Although individuals with obesity typically exhibit lower levels of physical activity, their higher resting energy expenditure is primarily explained by increased total body and fat-free mass. The enlargement of metabolically active organs and tissues, such as the liver, kidneys, heart, skeletal muscle, and even adipose tissue, contributes to elevated basal metabolic rate, independent of physical activity levels (35). Hence, the elevated REE in obesity seems to reflect a physiological adaptation to greater body mass, more aligned with the structural component of performance models than with the compensatory energy mechanisms observed in physically active individuals.

Components such as the metabolic and nervous systems have a disproportionately large impact on the increase in REE. Although they represent relatively small tissue masses, their high energetic cost accounts for a substantial part of the elevated REE. This increase is primarily attributable to the greater metabolic demands of vital organs, which must “keep pace” with overall body growth. Previous studies have shown that sex-related differences in body composition influence resting energy expenditure. In men, greater adiposity is often accompanied by additional muscle mass, which further contributes to REE, whereas women tend to preserve a higher proportion of metabolically active tissue relative to total body mass (31).

Although the increase in fat-free mass (FFM) partially explains the higher REE, evidence suggests that even after appropriate adjustments for body composition, REE remains significantly elevated in individuals with obesity, indicating additional compensatory metabolic mechanisms (34). The association between obesity and absolute energy expenditure largely reflects the increase in FFM in obese individuals. In this context, the rise in absolute energy expenditure may predominantly result from the greater volume of expansion of compartments with low metabolic rates, such as extracellular water and connective tissue (34).

Considering the relatively low metabolic cost of adipose tissue and its significant contribution to excess body mass, its expansion indirectly triggers adaptations that elevate energy expenditure (31). These include mechanical support adaptations in organs and systems (metabolically active tissues; brain/Central Nervous System, liver, heart, kidneys, skeletal muscle), as well as increased metabolic burden related to chronic inflammation, insulin resistance, and substrate turnover (31, 36). As a consequence, the higher REE observed in obesity may be more closely related to the metabolic demands of vital organs than to adipose tissue mass *per se* (31).

In our study, the difference of 324 kcal/day in REE is consistent with values reported in previous studies (31), not supporting the notion that adiposity indices are sustained by lower REE (37) or that REE cannot account for such differences (34). The average REE difference observed aligns more closely with values reported for class III obesity, as well as with values adjusted for FFM (34). This finding highlights that the relationship between obesity and metabolism is neither linear nor uniform, varying according to body weight status, sex, body composition, and degree of adiposity.

By challenging the commonly held notion that obesity is associated with a “slow metabolism” (38), our findings prompt further consideration of how the observed increase in energy expenditure is functionally regulated and directed. Despite greater fat mass, we suggest that, in obesity, the fasting metabolic profile is characterized by a higher relative contribution of lipids to total energy expenditure and a reduced capacity for carbohydrate oxidation. This metabolic adaptation may provide insights into how the body handles excess adiposity and how energy pathways are modulated across different BMI states.

Accordingly, resting lipid oxidation may reflect metabolic characteristics associated with obesity and could serve as a potential marker for discriminating metabolic profiles in adult populations. However, given the cross-sectional nature of our analysis, these variables should not be interpreted as causal predictors but rather as physiological markers of the obese phenotype. In this way, the resting lipid oxidation may reflect underlying metabolic characteristics associated with obesity and could serve as a potential marker for metabolic stratification rather than clinical categorization. The primary relevance of these findings lies in their potential to provide insights into energy substrate utilization patterns in obesity, which may guide the development of targeted lifestyle and therapeutic interventions aimed at improving metabolic flexibility, energy balance, and weight management. Future studies using longitudinal designs and detailed metabolic phenotyping could further define and validate these metabolic profiles, ultimately contributing to more accurate risk assessment and the personalization of preventive and clinical strategies.

Taken together, these factors may enhance the understanding of energy metabolism in obesity. Our findings reinforce the complexity of metabolic adaptations in this condition, in which higher absolute energy expenditure does not necessarily protect against fat accumulation, particularly in the presence of enhanced lipid oxidation. Such complexity underscores the importance of considering the physiological context when interpreting lipid metabolism markers across populations with different degrees of adiposity, ultimately improving healthcare professionals’ ability to address obesity and enhance quality of life.

### 4.3 Limitations

This study has some limitations. First, it was not possible to include male participants, which may limit the understanding of how obesity manifests across different genders. Second, we were unable to adjust for FFM, which could potentially underestimate REE values; however, the results were comparable to those reported in studies that performed such adjustments. Third, the lack of screening for recent weight loss constrains the assessment of potential metabolic adaptability. Fourth, we do not have specific clinical data (such as sleep apnea and medication use) to complement the analysis, and the absence of these data may limit the generalizability of the findings. Fifth, the lack of dietary control also represents a limitation. Although self-reported dietary data are often unreliable, we acknowledge that different dietary patterns, such as high-carbohydrate or low-carbohydrate, high-fat diets, may influence substrate oxidation. Furthermore, the number of participants in the groups was not similar, which may influence the results. Finally, our study did not account for other metabolic pathways that might also contribute to the observed alterations, and we acknowledge that interpretations based on BMI as hypotheses. We emphasize that BMI is a clinical screening index rather than a direct measure of adiposity or lean mass. We also recognize heterogeneity in the BMI as a limitation and how differences across obesity severity levels may influence resting REE results.

Despite these limitations, our study demonstrates methodological strengths that reinforce the robustness of its findings. The sample consisted of a relatively large and homogeneous group of women, allowing for the assessment of energy metabolism specifically in a female population, which is often underrepresented in metabolic research. The use of validated methodologies for indirect calorimetry and substrate oxidation ensured accurate assessment of resting energy expenditure and fuel utilization. Moreover, careful standardization of fasting and resting conditions minimized potential confounding factors. Overall, the methodological rigor, validated techniques, and well-characterized sample provide a solid foundation for interpreting our results. This study contributes to a better understanding of the relationship between energy metabolism parameters and susceptibility to obesity, offering physiological insights that may inform future lifestyle and therapeutic interventions aimed at improving metabolic efficiency and weight regulation.

## 5 Conclusion

The findings suggest that obesity is associated with alterations in energy metabolism, characterized by higher resting energy expenditure and lipid oxidation across BMI categories. These parameters may serve as indicators to differentiate individuals with obesity from those without, providing valuable insights for therapeutic interventions and prevention strategies.

Contrary to the assumption that obesity would lead to reduced fat mobilization, our data suggest that higher BMI, indicative of obesity, may be associated with a higher rate of resting lipid oxidation, possibly reflecting a chronic adaptation to greater adipose tissue mass, elevated circulating free fatty acids, and enhanced basal lipolysis. This underscores the role of body composition as a key modulator of resting substrate metabolism, suggesting that body composition directly influences fat mobilization at rest.

## Data Availability

The raw data supporting the conclusions of this article will be made available by the authors, without undue reservation.

## Footnotes

1
http://www.r-project.org/

## Supplementary material

Supplementary Figure S1 Distribution of BMI values across all participants (*N* = 216), categorized according to the WHO classification for normal weight, overweight, and obesity (Classes I–III).
