# Supplementary figures and images for: Energy metabolism and obesity stratified by BMI: impact on lipid oxidation, a cross-sectional observational study
Source: Front Nutr. 2025 Nov 27;12:1701686. doi: 10.3389/fnut.2025.1701686 (PMC12695580; doi:10.3389/fnut.2025.1701686)

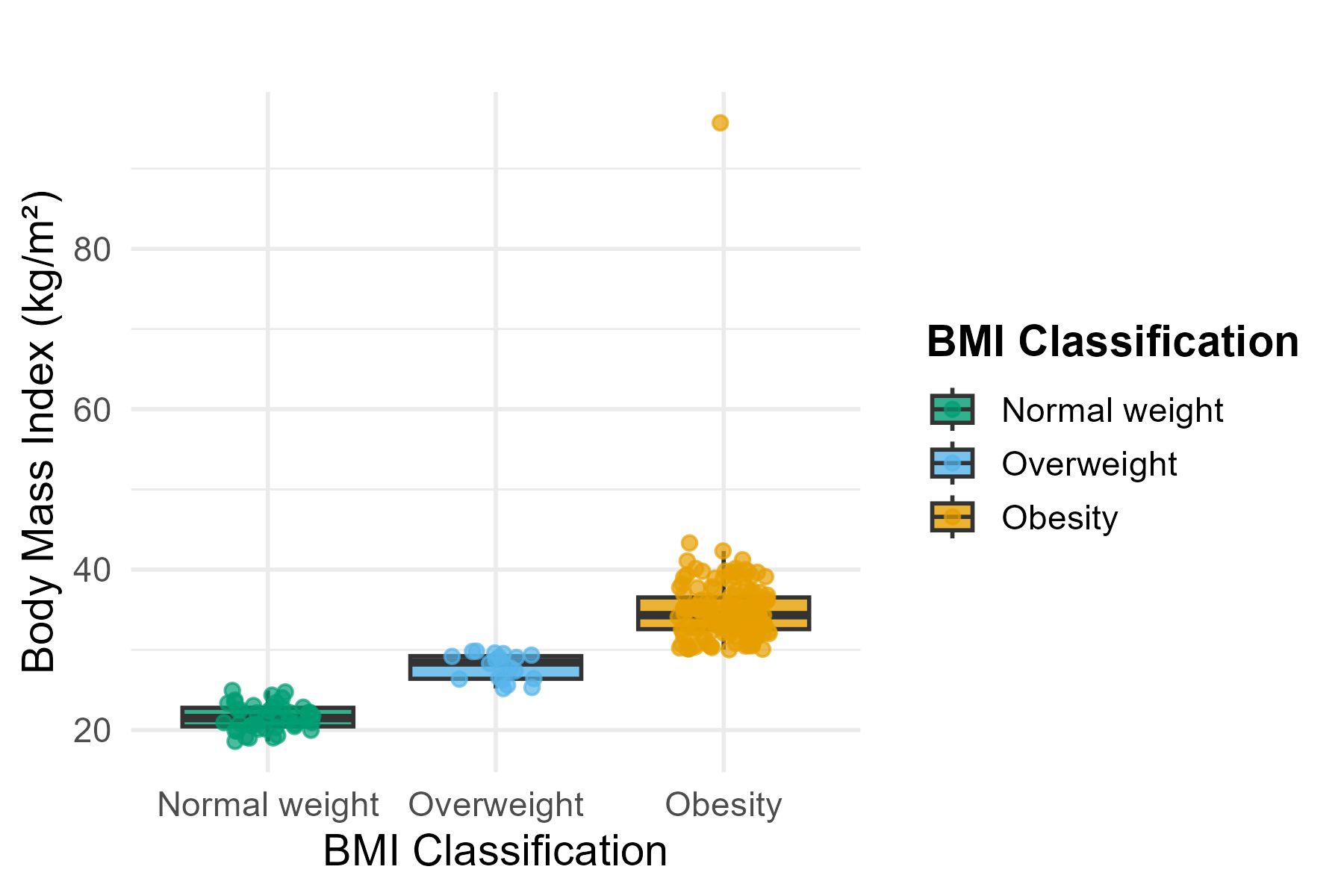

Supplement: Supplementary Figure S1 — Distribution of BMI values across all participants (N = 216), categorized according to the WHO classification for normal weight, overweight, and obesity (Classes I–III). [file Image_1.tiff]
