# Supplementary material for: Energy metabolism and obesity stratified by BMI: impact on lipid oxidation, a cross-sectional observational study
Source: Front Nutr. 2025 Nov 27;12:1701686. doi: 10.3389/fnut.2025.1701686 (PMC12695580; doi:10.3389/fnut.2025.1701686)
Supplement: Supplementary file 2 [file Table_1.pdf]

Reporting Guidelines Checklist – Metabolism (STROBE-based)

Manuscript title: Metabolism and Obesity stratified by BMI: Impact on Lipid Oxidation, a cross-sectional observational study

STROBE Item	Recommendation	Addressed in Manuscript (section)
**Title & Abstract**	Indicate the study design in the title and abstract; provide an informative and balanced summary.	Title specifies 'A cross-sectional observational study'; Abstract describes cross-sectional design (Abstract).
**Introduction – Background/rationale**	Explain the scientific background and rationale for the investigation.	Introduction
**Objectives**	State specific objectives, including hypotheses.	Abstract & Introduction
**Methods – Study design**	Present key elements of study design early.	Methods
**Setting**	Describe setting, locations, and dates.	Methods, Data sources & extraction.
**Participants**	Eligibility criteria, sources, and selection methods.	Methods
**Variables**	Define all outcomes, exposures, predictors, and confounders.	Methods: Indirect Calorimetry & Statistical Analysis.
**Data sources/measurement**	Explain how data were collected and measured.	Methods & Results
**Bias**	Describe efforts to address potential sources of bias.	Discussed indirectly under 'Limitations'.
**Study size**	Explain how sample size was determined.	Methods
**Quantitative variables**	Explain handling of quantitative variables.	Methods, Statistical analysis
**Statistical methods**	Describe methods, including subgroup analyses.	Methods, Statistical analysis
**Results – Participants**	Report numbers of individuals at each stage, with reasons for non-participation.	Methods
**Descriptive data**	Report characteristics of study participants.	Results, Table 1.
**Outcome data**	Report outcome events or summary measures.	Results, Tables 2–4 & Figures.

**Main results**	Present unadjusted and adjusted estimates with precision (e.g., CI).	Results, Tables 2–4.
**Other analyses**	Subgroup, sensitivity, interaction analyses if done.	ROC analysis, Results.
**Discussion – Key results**	Summarize key findings.	Discussion
**Limitations**	Discuss study limitations, bias, imprecision.	Limitations
**Interpretation**	Give cautious interpretation considering other evidence.	Discussion, Conclusions.
**Generalisability**	Discuss external validity of results.	Discussion, final paragraphs
**Funding**	Source of funding and role of funders.	Funding/Support
**Conflicts of interest**	Disclose conflicts of interest.	Declaration of competing interest
